# Successful treatment of acquired hemophilia A associated with immune thrombocytopenia and joint hemarthrosis: A case report and literature review

**DOI:** 10.1097/MD.0000000000012044

**Published:** 2018-09-21

**Authors:** Fang Wei

**Affiliations:** Department of Hematology, The First Hospital of Shanxi Medical University, Taiyuan, Shanxi, PR China.

**Keywords:** acquired hemophilia A, immune thrombocytopenia, joint hemarthrosis

## Abstract

**Introduction:**

Acquired hemophilia A (AHA) is a rare bleeding disease caused by autoantibodies against factor VIII (FVIII). Spontaneous bleeding symptoms usually affect the skin, musco, muscle, and internal organs, while joint hemarthrosis in AHA is an extremely rare manifestation. AHA may have an autoimmune cause and is often associated with autoimmune disease, but no demonstrable platelet impairment was found in AHA patients. We report a patient with AHA complicated with a right shoulder joint hemarthrosis and immune thrombocytopenia. The patient was treated with fresh frozen plasma (FFP) and human prothrombin complex concentrate (hPCC) to control the active bleeding. Simultaneously this patient firstly accepted cyclophosphamide combined with prednisone to eradicate the inhibitor, while the treatment effect of cyclophosphamide combined with prednisone was not satisfactory. At last, she was successfully treated through the use of an anti-CD20 monoclonal antibody.

**Conclusion:**

AHA is an autoimmune disease and can co-exist with immune cytopenia besides connective tissue disease (CTD). Joint hemarthrosis is not specific to congenital hemophilia and mainly related to the extent of prolonged aPTT and weight loading of joint in AHA. When the first-line therapy of cyclophosphamide combined with prednisone is not enough to eradicate the inhibitor, especially for a higher inhibitor titer, anti-CD20 monoclonal antibody could play an important role.

## 1 Introduction

Acquired hemophilia A (AHA) is a rare, potentially life-threatening autoimmune disorder caused by an autoantibody directed against factor VIII (FVIII). Unlike congenital hemophilia, AHA presents equally in men and women and occurs mainly in older patients than children, with the median age at presentation between 60 and 67 years.^[1]^ Bleeding patterns vary from superficial bruising that requires no hemostatic therapy to fatal internal bleeding, for example intracranial, retroperitoneal, gastrointestinal and lung bleeding.^[2]^ Patients with acquired hemophilia exhibit increased mortality.^[3]^ Although possibly associated with several underlying pathologies, nearly 50% of reported AHA cases remain idiopathic.^[2]^ AHA diagnosis is confirmed using special laboratory tests, and therapy is challenging due to the difficulty of diagnosis and comorbidities often associated with elderly patients. It has been reported that no demonstrable platelet impairment was observed in AHA patients,^[4]^ and spontaneous joint hemarthrosis is rarely in AHA cases.^[5]^

We report the first case of a patient with AHA complicated with joint hemarthrosis and immune thrombocytopenia who was successfully treated using an anti-CD20 monoclonal antibody.

## 2 Case report

A 40-year-old woman working as bank clerk was admitted to the Hematological Medicine division of our hospital. She presented with oral cavity and nose mucosal bleeding and muscle hematoma on both legs associated with homolateral painful knees that had lasted for 14 days. She denied any prior personal or family history of bleeding or clotting disorders. She had 2 artificial abortions, and one abdominal delivery without significant bleeding. Three days prior to the appearance of bleeding, she had been prescribed cephalosporin and proton pump inhibitors for 2 days due to abdominal pain and diarrhea. Upon admission, coagulation tests showed a severely prolonged activated partial thromboplastin time (aPTT) (107.4 seconds; reference value: 24.9–36.8 seconds) with normal prothrombin time (PT) and thrombin time (TT). Laboratory investigations revealed severe anemia (HGB: 49 g/L) and thrombocytopenia (PLT: 31 × 10^9^/L). Plasma bilirubin levels were normal and reticulocyte percentage was 1.4% (reference value: 0.5%–1.5%). Serum B12 and red cell folate concentrations were both within normal limits. Additional tests revealed that FVIII:C was 1% (reference value: 50%–150%), FIX:C was 130% (reference value: 50%–150%), FVIII inhibitor (FVIII:I) was 210 BU/ml (reference value: <0.6). The direct antiglobulin (Coombs) test was negative, platelet associated antibody IgG is positive, and Lupus anticoagulants (LAC) were negative. Complement levels were reduced (C4: 0.06 g/dL; reference value: 0.16–0.38 g/L, C3: 0.70 g/L; reference value: 0.79–1.52 g/L). Because this patient was a woman of child-bearing age who was likely to contract connective tissue disease (CTD), we also suspected she might be a CTD patient, particularly systemic lupus erythematosus (SLE). Therefore, we performed an autoimmune screen for CTD, including antinuclear antibodies, antidouble stranded antibody, extractable nuclear antigen (ENA) polypeptide antibody, antineutrophil cytoplasmic antibodies, antiphospholipid antibody and antithyroid antibody, all of which were normal. Virology tests for HIV, HPVB19, EBV, CMV, and hepatitis virus were all negative. A pectoral-abdominal computed tomography (CT) scan did not detect infection or tumor. In the end, the patient was diagnosed with AHA along with immune thrombocytopenia and blood-loss anemia. For treatment, the patient was initially transfused with packed red-blood cells to improve anemia, as well as with fresh frozen plasma (FFP) and human prothrombin complex concentrate (hPCC) to complement clotting factors. No additional hemorrhaging was observed; however, aPTT remained severely prolonged. As soon as the patient was diagnosed with AHA, she was administered intravenous immunoglobulin (IVIG) at a total dose of 2 g/kg over 5 days and started with oral prednisone at a dose of 1 mg/kg/day. Although the previous bleeding had subsided, and platelet counts gradually rose to near normal level after 2 weeks of treatment, the aPTT of this patient did not appear to shorten. Next, we administered intravenous cyclophosphamide (2.0 mg/kg/day) and continued oral prednisone at 1 mg/kg/day. While the aPTT of this patient remained severely prolonged, platelet counts reached a normal level. At week 3 of cyclophosphamide combined with prednisone treatment, the patient presented with moderate pain in the right shoulder region. An emergency CT was performed which showed the presence of a right shoulder joint hemarthrosis. We suspected the treatment effect of cyclophosphamide combined with prednisone was not satisfactory. We then administered intravenous anti-CD20 monoclonal antibody (Rituximab) treatment at 375 mg/m^2^ each week with tapering of the prednisone dose to 0.8 mg/kg/day. Following 2 weeks of anti-CD20 monoclonal antibody therapy, the prolonged APPT of this patient began to shorten and her shoulder joint bleeding gradually ceased. After 4 weeks of anti-CD20 monoclonal antibody therapy, both APTT and plasma FVIII were normalized, and the FVIII:I titer became undetectable. The duration of the course of anti-CD20 monoclonal antibody at 375 mg/m^2^ each week was 4 weeks. Prednisolone was gradually reduced at 10 mg each week until it was discontinued and the duration of prednisolone treatment was approximately 4 weeks. The patient remains in good medical status. There was no relapse during the 3-year follow-up period.

## 3 Discussion

### 3.1 Etiology of AHA

AHA results from the spontaneous emergence of autoantibodies directed against FVIII, which inhibit or neutralize FVIII. In addition an alloantibody to FVIII develops in 20% to 40% of congenital hemophilia patients who are treated with factor FVIII.^[6]^ Autoantibodies against FVIII occur in approximately 0.2–1.0 cases per million per year in nonhemophilia.^[7]^ The etiology of AHA is complicated and unknown; 46% of AHA patients are labeled idiopathic while more than 50% of cases are potentially associated with illnesses. The most commonly associated illnesses are autoimmune disease, such as as SLE and rheumatoid arthritis.^[8]^ The 2 largest case series showed an autoimmune association of 17% to 18%.^[8]^ Another frequently reported comorbidity is cancer or precancerous states, including solid tumors and lymphoproliferative malignancies.^[8,9,10]^ Skin disorders (such as pemphigus and epidermolysis bullosa), drugs (including penicillin, cephalosporin, and interferon), infections, postpartum and chronic graft-versus-host disease have also been reported to be associated with AHA.^[8,9,11,12]^ Because the patient in our case study was a woman of child-bearing age and plasma complement C3 and C4 were reduced, a diagnosis of CTD, especially SLE, could not be excluded. However, the results of multiple laboratory tests for CTD were negative and the patient lacked typical CTD symptoms, therefore CTD could not be diagnosed. The work-up of malignancy was also unremarkable. Given her use of cephalosporin prior to bleeding symptoms, we suspected an association between cephalosporin and AHA in this patient, but this could not be definitively identified.

### 3.2 Clinical presentation of AHA

Almost 90% of AHA cases manifest as severe hemorrhagic diathesis. Extensive subcutaneous blood extravasations, mucosal hemorrhages, bleeding from postoperative surgical wounds and after tooth extraction procedures, and painful intramuscular hematomas are all typical in AHA patients. In contrast to congenital hemophilia, spontaneous joint hemarthrosis is rarely observed in AHA cases. However, our patient presented with shoulder joint hemarthrosis. We suspect the joint hemarthrosis in this patient was the result of severe prolonged aPTT. Therefore, we consider that joint hemarthrosis was related to the extent of prolonged aPTT and weight load in the joint and believed that joint hemarthrosis is not specific to congenital hemophilia.

### 3.3 Diagnosis of AHA

Although rare, AHA should be considered in differential diagnosis, particularly in women and the elderly with bleeding tendency or prolonged aPTT. When a patient without a hematologic history presents with prolonged aPTT, PT and a suggestive clinical picture, AHA should be suspected. The most common way to delineate the cause of the prolonged aPTT is to perform a mixing study after heparin contamination is excluded. If aPTT is corrected, this suggests a clotting factor deficiency, and if not, an inhibitor to the factor is likely. The latter can be a lupus anticoagulant or an inhibitor to the clotting factor, commonly FVIII. If only one clotting factor is reduced by the inhibitor, this would suggest the presence of the lupus anticoagulant factor where antibodies can affect the laboratory estimation of more than one factor. The strength of an inhibitor can be quantified using the Bethesda assay, which measures residual FVIII activity after incubation of normal plasma with serial dilutions of patient plasma for 2 hours at 37°C. The inhibitor titer in BU represents the reciprocal of the dilution of the patient's plasma which leads to 50% inhibition in the assay. The patient's clotting test result was consistent with the diagnostic criteria for AHA with a negative autoimmune screen. Given this, the patient was diagnosed as having AHA. Anemia is also common in AHA patients, but the cause of anemia is rarely reported. Since this patient also presented with severe anemia on admission, we performed laboratory tests for anemia, and noted that serum B12 and red cell folate concentrations were within normal limits. Reticulocyte percentages and serum bilirubin were normal, and the Coombs test was negative. The extent of the patient's anemia was not further aggravated once the active bleeding was controlled. Therefore, we strongly suspect anemia in this patient was the result of blood loss, and not hemolysis.

It has been reported that no demonstrable platelet impairment is observed in AHA patients.^[4]^ However, the patient in our study did present with severe thrombocytopenia. Further laboratory test confirmed that the platelet associated antibody IgG of this patient was positive, and the platelet count could be recovered using immunosuppressant therapy. Therefore, this patient was simultaneously diagnosed with AHA and immune thrombocytopenia. We inferred that autoantibodies could be simultaneously directed against FVIII and platelets in this patient, resulting in deficiency of FVIII and platelets. This data shows that AHA potentially accompanies other autoimmune disorder besides CTD, which increases the difficulty of diagnosis of the disease and leads to significant mortality.

### 3.4 Therapy of AHA

The mortality rate of AHA is between 7.9% and 22%.^[1]^ Once a patient is diagnosed with AHA, appropriate therapy directed at arresting hemorrhage and eradicating the inhibitor must be initiated. If plasma FVIII levels can be increased to 30% to 50% in a patient with AHA, hemostasis can generally be achieved.^[1]^ This result can be achieved using either DDAVP or infusion of FVIII. If a patient has severe bleeding and an inhibitor titer >5BU, it is prudent to begin therapy with an agent that bypasses FVIII, either activated prothrombin complex concentrate (aPCC) or recombinant factor VIIα (rVIIα).^[1]^ Aside from treating the bleeding, removal of the inhibitor is critical to achieving long term, stable AHA control. Presently, immunosuppressive medication plays the most crucial role in inhibitor elimination. Immunosuppressive agents include corticosteroids (prednisone 1 mg/kg/day), cyclophosphamide (1.5–2.0 mg/kg/day), IVIG (total dosing of 2 g/kg over 2 or 5 days), cyclosporine A (4–6 mg/kg/day),^[13]^ and anti-CD20 monoclonal antibodies, which can be used either alone or in combination with other medication. The optimal immunosuppressive regimen is unclear. In the last several years, anti-CD20 monoclonal antibody has been used for the management of various autoimmune diseases, as well as lymphomas. “Off label” use of anti-CD20 monoclonal antibody has been studied for patients with acquired hemophilia, showing promising results of durable remission.^[14–16]^ The usual dose is 375 mg/m^2^ each week for 4 weeks. Most responses are observed within the first 2 weeks of therapy, with some clinician's inputs, it should be considered in patients resistant to first-line therapy or for those that cannot tolerate standard immunosuppressive therapy.^[9]^ FVIII:I titer for the patient in the current study was 210 BU/mL, which is much more than 5 BU/mL, therefore the patient was initially transfused with FFP and hPCC to complement clotting factors for treatment of bleeding, when FVIII, aPCC and VIIα were unavailable. At the same time, this patient initially accepted corticosteroids combined with IVIG or intravenous cyclophosphamide to eradicate the inhibitor. After 1 month of corticosteroids-based combination treatment, the aPTT of the patient remained severe prolonged. The patient then presented with shoulder joint hemarthrosis, which was her second episode of bleeding. As a result of the very high inhibitor titer and severe bleeding presented by this patient, we adopted an anti-CD20 monoclonal antibody treatment plan at 375 mg/m^2^ each week combined with an oral dose of prednisone at 0.8 mg/kg/day. Following 4 weeks of anti-CD20 monoclonal antibody therapy, the patient achieved complete remission. Although rituximab-based regimens showed good results, the current study did not support the use of rituximab alone or, if used as a first-line treatment, which is a better or safer option than other less expensive immunosuppressive agents.^[17]^

In conclusion, we present a very rare account of a patient with AHA complicated by joint hemarthrosis and immune thrombocytopenia which was successfully managed. We learned that AHA is an immune disease and AHA can co-exist with immune cytopenia in addition to CTD, which often makes patient presentation complicated and increases the difficulty of accurate diagnosis and therapy. As hematological physicians, we should acquire more knowledge on AHA and associated autoimmune diseases, recognize the shared pathophysiology of these associated autoimmune conditions, and make prompt and accurate diagnoses. When the first-line therapy using cyclophosphamide combined with prednisone is not enough to eradicate the inhibitor, especially for a higher inhibitor titer, anti-CD20 monoclonal antibody therapy could be beneficial in these patients.

## Acknowledgments

We gratefully acknowledge the patient and his family members. Patient consent was obtained.

## Author contributions

**Funding acquisition:** Fang Wei.

**Writing – original draft:** Fang Wei.

**Writing – review & editing:** Fang Wei.
